# Neonatal Meningitis: Risk Factors, Causes, and Neurologic Complications

**Published:** 2014

**Authors:** Nasrin KHALESSI, Ladan AFSHARKHAS

**Affiliations:** 1 Division of Neonatology, Ali-Asghar Children’s Hospital, Iran University of Medical Science, Tehran, Iran.; 2 Department of Pediatric Neurology, Ali-Asghar Children’s Hospital, Iran University of Medical Science, Tehran, Iran.

**Keywords:** Neonatal meningitis, Risk factor, Complication

## Abstract

**Objective:**

Neonates are at greater risk for sepsis and meningitis than other ages and in spite of rapid diagnoses of pathogens and treatments, they still contribute to complications and mortality. This study determines risk factors, causes, and neurologic complications of neonatal meningitis in hospitalized neonates.

**Material & Methods:**

In this descriptive, cross sectional study, we evaluated 415 neonates with sepsis and meningitis admitted to the neonatal intensive care unit at our center between 2008 and 2012. The data that was recorded was age, sex, birth weight, prenatal risk factors, clinical features, blood and cerebrospinal fluid analysis, and brain sonographic findings and outcomes.

**Results:**

Twenty patients had meningitis. Eleven cases (55%) were male. The mean age was 8. 41 days and mean birth weight was 2891.5±766 grams. Poor feeding, seizures, and tachypnea were detected in 12 (60%), 11 (55%), and 6 (30%) patients, respectively. Prenatal risk factors were prolonged rupture of membranes, maternal vaginitis, asymptomatic bacteriuria, prematurity, low birth weights, and asphyxia. Four patients had positive cerebrospinal fluid cultures with klebsiella pneumoniae 2 (50%), Enterococcus spp. 1 (25%), and Group B streptococcus 1 (25%) cases, respectively. Two cases had positive blood cultures with klebsiella pneumoniae. Neurologic complications were brain edema, subdural effusion, and brain abscesses with hydrocephaly. One neonate (5%) died.

**Conclusion:**

Our study provides some information about risk factors, pathogens, and neurologic complications for neonatal meningitis. Prenatal assessments help to diagnose and reduce risk factors of this hazardous disease.

## Introduction

In spite of the development of the rapid diagnosis of pathogens and new antibiotics, neonatal meningitis (NM) contributes to neonatal mortality and morbidity worldwide. Neonatal meningitis is the inflammation of the meninges during the first 28 days of life (1). According to the time of diagnosis, it is classified as earlyonset (EOM) or late onset meningitis (LOM). In EOM, clinical features appear during the first weeks of life. LOM occurs between 8–28 postnatal days (2,3). The incidence of neonatal bacterial meningitis ranges from 0.25 to 1 per 1000 live birth and occurs in 25% of neonates with bacteremia (4,5). In developed countries, group B streptococci (GBS) are the most common causes of bacterial meningitis, accounting for 50% of all cases. Escherichia Coli (E. Coli) accounts for another 20%. Thus, identification and treatment of maternal genitourinary infections is an important prevention strategy (6). In developing countries, gramnegative bacilli such as Klebsiella and E. Coli may be more common than GBS especially in LOM (7,8). In addition, other organisms that have been implicated as a cause of meningitis include Enterobacter spp., Citrobacter spp., and serratia spp. Meningitis is often more severe with gram-negative bacteria and with a higher rate of mortality and morbidity (9). Diagnosis of NM is based on both clinical manifestations and cerebrospinal fluid (CSF) examination. CSF culture is an excellent exam for demonstration of meningitis. Evaluation of leukocyte count, glucose, and protein levels in the CSF may help in the diagnosis (10). This study evaluates neonates who were admitted with meningitis from 2008 to 2012 in our tertiary center. We evaluated maternal and neonatal risk factors, clinical manifestations, pathogens, and neurologic complications of neonatal meningitis cases. 

## Materials & Methods

In this retrospective cross-sectional study, we included all admitted neonates with diagnosis of sepsis and meningitis in Ali-Asghar Training Children’s Hospital, Tehran, between 2008 and 2012. Medical records of the neonates who were referred to our center for a sepsis work up were investigated. Infants older than 28 days, congenital infections (TORCHs syndromes), central nervous system anomalies, and severe intraventricular hemorrhage were excluded. Lumbar punctures were routinely performed on every patient as part of the sepsis work up before starting empirical antibiotics to rule out meningitis. A definitive diagnosis of meningitis was based on the growth of a pathogen from primary CSF culture and supportive clinical manifestation (seizures, thermal instability, and feeding intolerance, among others). Suspected meningitis was diagnosed if no organism was obtained from the CSF culture and CSF characteristics (Leukocyte count > 32/mm3 and > 29/mm3; glucose level < 34 mg/dl and < 24 mg/dl; and protein level > 170 mg/dl and > 150 mg/dl as the criteria of meningitis in term and premature neonates, respectively) was suggestive for NM (11). Gestational age (GA), gender, birth weight, onset of infection (EOM, LOM), risk factors, clinical findings, CSF analysis, CSF, and blood cultures were recorded. Risk factors such as prolonged rupture of membranes (> 18 hours), mother’s vaginitis and asymptomatic bacteriuria, prematurity (GA <37 week), low birth weight (LBW; i.e. a birth weight <2500gr), multiple birth pregnancy, and asphyxia were investigated. Clinical findings such as fever, poor feeding, seizures, and tachypnea were noted.

Laboratory finding such as CSF analysis, blood, and CSF cultures were evaluated. All patients with meningitis were evaluated by brain sonography. Neonates with an abnormal brain sonography were examined with brain magnetic resonance imaging (MRI). Mortality rate was recorded. Data were analyzed using SPSS (ver. 14). This study was approved by ethics committee of the Iran University of Medical Science.

## Results

Between April 2008 and August 2012, out of 415 neonates were hospitalized for sepsis work up, 20 patients (4–8%) were diagnosed with meningitis. A total of 55% of the patients were male. A total of 16 patients (80%) were term. The mean birth weight was 2891.5±766 grams. A total of 13 patients (65%) had EOM and 7 patients (35%) had LOM. Maternal risk factors were included PROM, mother’s vaginitis during third trimester, and asymptomatic bacteriuria. Neonatal risk factors for NM were prematurity, LBW, multiple birth pregnancy, and asphyxia (Table-1). linical findings included poor feeding 12 (60%), seizures 11 (55%), tachypnea 6 (30%), fever 6 (30%), decreased Moro reflex 5 (25%), irritability 2 (10%), icter 2 (10%), bulging of fontanelle 1 (5%) of cases. The mean CSF leukocyte count was 741±473 cells/mm3. Mean glucose and protein levels in CSF were 49±39 mg/dl and 120±93 mg/dl, respectively. CSF cultures were positive in 4 (20%) patients and pathogens included klebsiella pneumoniae, GBS, and Enterococcus spp (Table 2). Blood cultures were performed for all patients and two patients had positive blood cultures with klebsiella pneumoniae with the same results in CSF cultures (Table 2). 

All patients with meningitis were evaluated by brain sonography. The brain sonographies revealed abnormal findings as early neurologic complications in four cases (20%) including hydrocephaly with abscess formation, solely hydrocephaly, subdural effusion, and brain edema (Table -3). A brain MRI was performed for patients with an abnormal brain sonography and each was confirmed. The mortality rate was 5%. This case was a term and male neonate from a difficult vaginal delivery and was resuscitated at the first minutes of life and had seizures along with refractory to antiepileptic therapy. Cerebrospinal fluid analysis after one week of hospitalization, showed abnormal finding including WBC=450/mm3 (segmented cell = 40%), RBC = 3/ mm3, glucose =4mg/dl, protein =230mg/dl, and serum glucose at the same time was 85mg/dl. Blood and CSF culture were negative. Severe brain edema revealed by brain sonography and MRI. The patient died on day 55 from his birthdate.

## Discussion

In this study, we identified 20 cases of neonatal meningitis. Our data indicated that NM is found predominantly in boys and our results confirmed the findings of Laving et al and Kavuncoglu et al (2, 14). They have mentioned that there is a gender–linked susceptibility to meningitis. 

In our survey, according to reported age of the patients at the time of admission (in their medical records), 65% of neonates were younger than 7 days as EOM and it is similar to the findings of Chang et al, which among 85 patients treated with diagnosis of NM, 51 (60%) had EOM. It is worth mentioning that the prevalence of NM is higher in late onset sepsis but, according to our findings, maternal risk factors that contribute to EOM were more common in our patients. It is suggested to place greater attention on maternal and prenatal care especially during the third trimester to early diagnosis and treatment of genitourinary infections. 

According to this study, maternal risk factors for NM were PROM, mother’s third trimester vaginitis, and asymptomatic bacteriuria. In a study in Turkey, PROM was involved in 12.5% of cases with EOM and 8.6% of these patients had positive bacterial growth in maternal urinary or cervical cultures (14). Neonatal risk factors of meningitis in our study were prematurity, LBW, asphyxia, and multiple birth pregnancy, which was similar to Gerdes et al (15). 

The presenting signs and symptoms of NM are nonspecific. Common symptoms include poor feeding, lethargy, vomiting, respiratory distress, and temperature instabilities (11). In our study, the most common clinical manifestations were poor feeding, seizures, and tachypnea.

In our study, CSF culture confirmed meningitis contributed to 20% of the cases and klebsiella pneumoniae were the most commonly detected pathogens. Other pathogens were GBS and Enterococcus spp. In Aletayeb, which was similar to our study, klebsiella pneumoniae was the common cause of EOM and LOM. Other isolated pathogens were Enterobacter, E.coli, Enterococcus spp., Pseudomonas aeruginosa, and Staphylococcus aureus. With this proportion, it is suggested to pay greater attention to other pathogens than GBS in prenatal cares in our country. In our investigation, the rate of positive CSF culture was lower than other references. It may be due to maternal antibiotic prophylaxis or delayed LP in antibiotic treated neonates and in these situations, clinicians have to rely on the CSF parameters specially WBC and clinical features to determine NM (10).

In this study, positive blood cultures were detected in 2 cases (50%) of patients that had positive CSF cultures with the same microorganism (klebsiella pneumoniae). It is suggested that meningitis frequently occurs in the absence of bacteremia and lumbar punctures as an important part of the diagnostic evaluation in suspected cases (10). A total of 58% of the NM cases in Aletayeb et al had a positive blood culture for the same organisms isolated from the CSF (2). A positive blood culture in their study was higher than for our study and this difference may be due to a higher rate of antibiotic prescribing during labor and a lower quality of culture techniques at our center. 

**Table1:** Characteristics of Neonatal Meningitis Cases

**variables**	**Early-Onset** (< 7days) n=13 No.(%)	**Late –Onset** (> 7days) n=7 No. (%)
**Sex** M F	9(45%) 4(20%)	2(10%) 5(25%)
Neonatal RF*		
Prematurity LBW Asphyxia Multiple birth	4(20%) 4(20%) 2(10%) 2(10%)	none
**Maternal RF**		
PROM Asymptomatic bacteriuria Vaginitis	2(10%) 1(5%) 1(5%)	
Mortality	none	1(5%)

RF: Risk Factor

**Table 2:** Results of CSF and Blood Cultures

**Pathogens**	**No.(%)**
**CSF Culture**	
Enterococcus spp. GBS Klebsiella pneumoniae	1(25%) 1(25%) 2(50%)
**Blood culture**	
Klebsiella pneumoniae	2(100%)

**Table 3:** Neurologic Complications Observed in Brain Sonography

**Brain sonography findings**	**No. (%)**
Hydrocephaly Hydrocephaly and Abscess formation Brain edema Subdural Effusion	1(25%) 1(25%) 1(25%) 1(25%)

In the present study, neurologic complications were detected in 20% of cases in brain sonography, including hydrocephaly with and without abscess formation, subdural effusion, and brain edema. A brain MRI confirmed our data and neurosurgical consultations were performed to evaluate these cases. In Kavuncoglu et al, pathological sonographic findings were ventricular dilatation, hydrocephalus, and intracranial hemorrhage 14). It is recommended that brain sonography be performed as a baseline study in every infant with suspicion of bacterial meningitis. In patients with complicated bacterial meningitis, an MRI should be the next study of choice (16).

In our study, the mortality rate was 5%, because of the association of asphyxia with NM. In Aletayeb et al, the mortality rate was 30%. This difference may be due to higher rate of low birth weight (60%) in their study, which has an important role in mortality (2). 

There were some limitations in our study including a small sample size and lack of technical facilities for a culture of viruses in suspected cases of nonbacterial meningitis.

**In conclusion, **we found that in our center, EOM was more common than LOM and klebsiella pneumonia was the most common cause of neonatal meningitis. Hydrocephaly, brain edema, and subdural effusion were neurologic complications of patients with NM. More emphasis on prenatal assessments may reduce maternal risk factors and neurologic complications of this hazardous disease.
